# Food security and welfare changes under COVID-19 in Sub-Saharan Africa: Impacts and responses in Kenya

**DOI:** 10.1016/j.gfs.2021.100514

**Published:** 2021-03

**Authors:** Victor Nechifor, Maria Priscila Ramos, Emanuele Ferrari, Joshua Laichena, Evelyne Kihiu, Daniel Omanyo, Rodgers Musamali, Benson Kiriga

**Affiliations:** a European Commission, Joint Research Centre (JRC), Seville, Spain; b Universidad de Buenos Aires, Facultad de Ciencias Económicas, Departamento de Economía, CONICET-Universidad de Buenos Aires, Instituto Interdisciplinario de Economía Política de Buenos Aires (IIEP-Baires), Ciudad Autónoma de Buenos Aires, Argentina; c Kenya Institute for Public Policy Research and Analysis (KIPPRA), Nairobi, Kenya

**Keywords:** COVID-19, Economy-wide analysis, Food sufficiency, Nutrition, Sub-Saharan africa

## Abstract

The COVID-19 pandemic has affected all Sub-Saharan economies through a multitude of impact channels. The study determines the medium-term macroeconomic outcomes of the pandemic on the Kenyan economy and links the results with a detailed food security and nutrition microsimulation module. It thus evaluates the effectiveness of the adopted government measures to reduce the negative outcomes on food security and to enable economic recovery at aggregate, sectoral and household levels. Through income support measures, the food sector and food demand partially recover. However, 1.3% of households still fall below calorie intake thresholds, many of which are in rural areas. Results also indicate that the state of food security in Kenya remains vulnerable to the evolution of the pandemic abroad.

## Highlights

• Economic and food security COVID-19 impacts assessed with a macro-micro framework.
• The food sector and food demand partially recover through income support measures.
• Rural households have the lowest recovery in food sufficiency and adequacy levels.
• Food security outcomes remain exposed to the evolution of the pandemic abroad.
• More household income support is required in case of further restrictions.

## 1 Introduction

The COVID-19 pandemic will likely generate one of the deepest global economic recessions in decades with the world economy potentially taking a few years to recover back to its pre-COVID-19 levels. Declared as a pandemic by the WHO in mid-March 2020, the economic effects of COVID-19 on the African continent may have been felt sooner than the occurrence of the first cases. The strict lockdown measures taken in China at the beginning of the year, followed by further restrictions in Europe and the USA, have taken their toll on the international movement of goods and people, with significant impacts on international trade and tourism. There are early indications that global trade has already fallen by 5% in the first quarter of 2020 and is set to decline by 20% over the year (UNCTAD, 2020a).

In addition to the immediate health concerns, the pandemic is affecting food systems globally and has negative impacts on all four pillars of food and nutrition security: availability, accessibility, utilisation, and stability (Laborde et al., 2020a). Given the strong positive correlation between economic recession and food insecurity in Africa, COVID-19 threatens access to food mainly through losses of income and assets, thereby jeopardizing the possibilities and capacities to buy food. Impacts are also felt through disruptions to availability; shifts in consumer demand toward cheaper, less nutritious foods; and food price instability (Laborde et al., 2020b). The measures to prevent the spread of the pandemic affect African economies under many aspects, for example, reducing both public revenue and merchandise exports which are expected to contract by about 17% in 2020 (UNCTAD, 2020b). With at least 50% of the African population dependent on agriculture for their livelihoods and access to food (AfDB, 2016; World Bank, 2020a), any trade-related distortions threaten the food security of many African countries and their progress towards the Sustainable Development Goals (SDGs).

During the first months of the pandemic, several assessments of impacts on global, regional and national economic systems, food systems, food security and agricultural markets have been produced. A preliminary assessment provided by Maliszewska et al. (2020) finds that countries in Sub-Saharan Africa (SSA) and the Middle East and North Africa (MENA) are the least affected, and under the global and amplified global pandemic scenarios, the estimated loss of GDP is around 3%. At the same time, compared to the world average, least developed countries (LDCs) will show a higher demand reduction in all food sectors (Gay et al., 2020), while in all countries the impacts will be larger for the poorest segments of the population. A global macroeconomic approach (Laborde et al., 2020b) suggests that following a projected downturn in global economic growth of 5% in 2020, Africa will be hit harder with a decline of around 9%. While agri-food sectors may expand as the collapse in export earnings and loss of capacity to import food push up domestic production, lower labour demand in urban service sectors push workers towards agriculture contributing to increased food production. Nevertheless, these quantifications relying on global modelling lack the ability to specify national and sub-national level characteristics, especially for smaller economies, and thus tend to over-simplify impact channels.^1^

Single country analyses based on Social Accounting Matrix (SAM) multiplier models have estimated the economic costs of COVID-19 in sub-Saharan countries such as South Africa (Arndt et al., 2020), Ghana (Amewu et al., 2020) and Malawi (Baulch et al., 2020). While these country-level analyses include details on how the food sector output and prices could be impacted by confinement during the pandemic, to the best of our knowledge, there is no evidence related to how these impacts are translated into food security changes at the household level. Given the very short-term focus of these assessments of one or two quarters, they are able to consider the timing of lockdowns in relations to the annual cycle of seasonal labour demand which is important in agriculture-dominated economies (Feuerbacher et al., 2020). Nevertheless, the evidence provided does not offer a view on household and production adaptation through substitution possibilities nor on income and food demand dynamics once the lockdown measures are eased and when households continue to be impacted by the aggregate reduction in economic activity.

Kenya is a case in point for the short-term pandemic recovery in Sub-Saharan Africa from a number of viewpoints: malnutrition in the general population remained a challenge even prior to the pandemic (FAO, 2020), agriculture and informal activities represent a large share of the economy, the export of commodities represents a significant source of foreign currency, many households are dependent on remittances (World Bank, 2020b), and the government's fiscal space to tackle the effects of the pandemic is limited (UNU-WIDER, 2020).

Prior to the COVID-19 pandemic, the food poverty incidence in Kenya remained high as about 1 in every 3 individuals did not meet the minimum daily calorific requirement of 2,250 kilocalories as per their expenditures on food (KNBS, 2018). Food poverty was higher in rural areas where 35.8% of the population (10.4 million individuals) live below the food poverty line, much higher compared to 28.9% (0.8 million individuals) in peri-urban areas and 24.4% (3.7 million individuals) in core-urban. Consequently, tackling food security and nutritional impacts due to COVID-19 disruptions has become paramount and the government has implemented a set of public spending and fiscal measures to mitigate the impacts of the pandemic on the economy and on households’ income.

This article evaluates the implications of COVID-19 pandemic on the Kenyan economy and on food security in 2020. The analysis considers several impact channels of the pandemic taken alone - labour productivity, export demand and tourism, remittances, internal demand and internal trade costs. It then determines the effectiveness of government measures to enable economic recovery and to reduce the negative outcomes of the pandemic on food security by characterising impacts at different levels: aggregate (GDP, employment and trade), sectoral (production levels) and household (income, consumer demand, food sufficiency and adequacy). The assessment includes the uncertainty of lockdown durations both domestically and abroad and incorporates the Kenyan government fiscal and spending measures implemented through the Tax Laws (Amendment) Act, 2020, the COVID-19 Spending Plan and the Economic Stimulus Plan.

By accounting for the complexity of interactions between the impacts and their incidence across all areas of the economy (households, farms, enterprises and government), this study summarises and updates the economy-wide analysis in Nechifor et al. (2020) and links the results with a detailed food security and nutrition microsimulation module based on the latest Kenyan Integrated Household Budget Survey (KIHBS), 2015–2016.

The rest of the paper is organised as follows. The next section introduces the Kenyan measures to contain the pandemic and its possible impacts on the economy and food security. It then elaborates on impacts of COVID-19 measures on Kenya's food security through different impact channels. Section 3 describes the toolset used for the analysis together with the COVID-19 scenarios considered. Section 4 records the main results in terms of macroeconomic effects, household welfare, and food sufficiency and food adequacy. The last section discusses the policy implications of the simulation analysis.

## 2 Impacts of COVID-19 measures on Kenya's food security through different impact channels

**Fig. 1:**
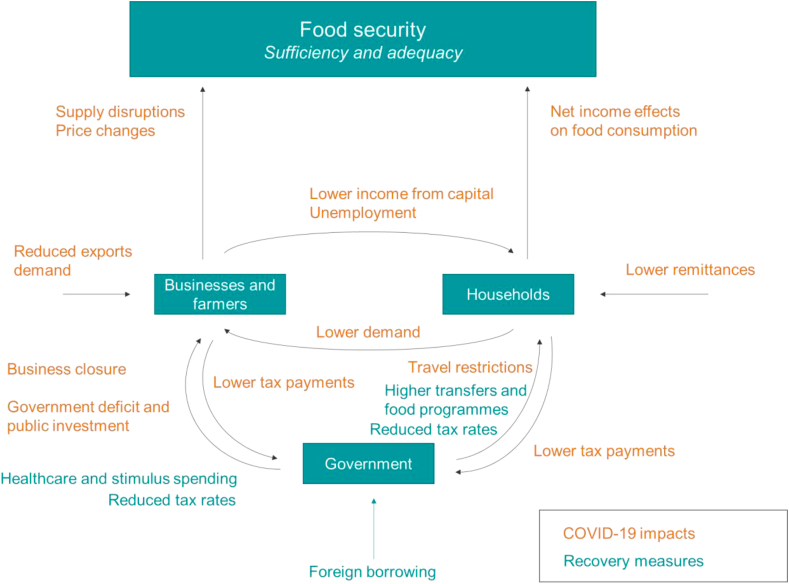
Interactions between COVID-19 lockdown impacts and government recovery measures leading to food security outcomes.

Source: Authors

Food security is tightly connected to the evolution of household income during and after the lockdown (the demand-side effect), and the capacity of food systems to produce foodstuff under the constraints of social distancing along the food supply chain (the supply-side effect) (Fig. 1). Therefore, considering the complexity of interactions between the COVID-19 lockdown impacts and government measures, the analysis of household income and food security under the pandemic pleads for an economy-wide perspective. Given that agriculture in Kenya is central to the economy (it produces a third of the country's GDP and employs more than half of the labour force (World Bank, 2020a), and that Kenyan households dedicate a large share of their disposable income to food (33% in urban areas and 48% in rural areas (Mainar Causapé et al., 2018, p. 23), an economy-wide approach to food systems analysis becomes even more relevant.

With the declaration of the pandemic as a Formidable Epidemic Disease on 27^th^ of March 2020, the Kenyan government introduced a set of restrictions and social-distancing protocols including: (a) closure of educational institutions, (b) suspension of international flights except cargo and evacuation planes with an imposition of a 14-day quarantine for returning residents, (c) reduction of public transportation capacity to below 60%, (d) suspension of domestic flight and passenger railway train, (e) recommendation for people to stay and work from home and the banning of public gatherings including places of worship, hotels, bars and restaurants, (f) movement restrictions from and to the counties of Nairobi, Kwale, Kilifi, Mandera and Mombasa and (g) requirement for all persons to wear face masks while in public places.

Under this lockdown, household income was affected directly through a contraction of economic activity from full or partial business closure leading to lower earnings from employment and other rents. At its turn, the lower household income may have translated into lower demand across the consumption basket with further negative feedback effects on economic activity. The evolution of the pandemic outside the country also impacted the economy in multiple ways. The reduction in commodity and service demand in other countries determined a contraction of exports and tourism with severe implications over the activity of export-oriented sectors such as horticulture. The potential reduction in employment of the diaspora also risked reducing household income through lower remittances. The implied reduction of foreign exchange inflows determined a depreciation of the local currency and may have determined an increase in relative prices of imported goods.

In response to these negative effects, the Kenyan government adopted a series of recovery measures. It intervened to influence the prices of commodities and implicitly food through changes in VAT taxation, and sought to support domestic production through a reduction in taxation (e.g. turnover and dividend taxes) and to boost household income through increases in household transfers and reductions in income taxation. The government equally put in place a set of measures to address the healthcare crisis and support the recovery of economic activities. A budget of KSh 44.8 billion (0.46% of Kenya's GDP) was allocated for increased healthcare spending and COVID-19 monitoring costs and for enhanced social protection, cash transfers and food relief programmes. The government also announced a further KSh 37.7 billion under the Economic Stimulus Programme dedicated to infrastructure development and sector support for agriculture, manufacturing and tourism. All these measures do come at a cost as the government budget is impacted both on the revenue side, given lower tax receipts from a contracted economy, and on the spending side as public expenditure increases.

## 3 Methods

### 3.1 Macro-micro analysis framework

**Fig. 2:**
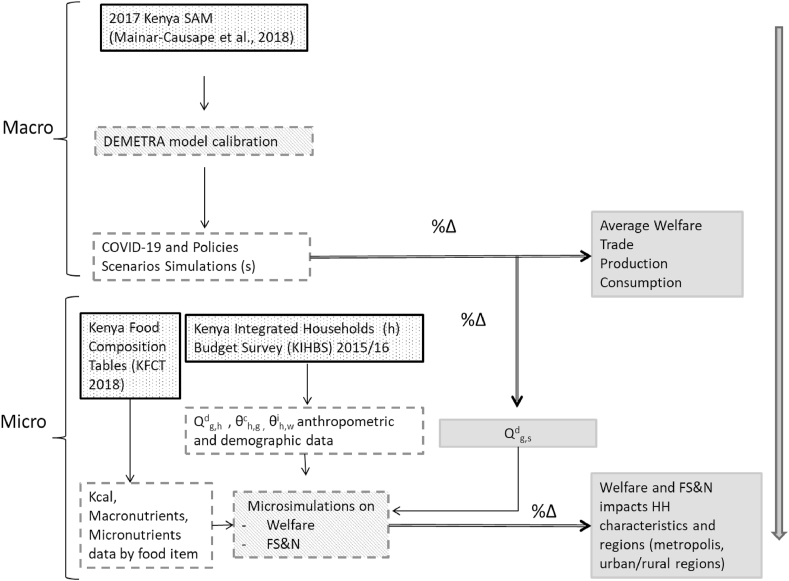
Macro-micro model coupling for determining FS&N impacts.

Source: adapted from Ramos et al. (2020).

To evaluate the implications of the above impact channels on food security, this analysis uses an economy-wide model for the Kenyan economy (a computable general equilibrium (CGE) model) integrated with a Food Security and Nutrition (FS&N) microsimulation module calibrated using the latest KIHBS of 2015/2016. In the macro-micro coupling adopted as in Ramos et al. (2020), changes in aggregate household food demand computed by the CGE model are passed on to the microsimulation module to determine FS&N impacts across the food sufficiency and food adequacy metrics and across different household characteristics (per capita expenditure, per capita calorie intake and presence of stunting in households’ children) - Fig. 2. This coupling thus enables the calculation of detailed economy-wide effects at a sectoral and sub-national level with a further expansion of COVID-19 FS&N impacts across the full range of household income percentiles.

The study employs the DEMETRA model^2^ for Kenya, a single-country CGE model which comprises a large number of economic sectors and households. The model results therefore allow for an advanced characterisation of impacts at different levels: sectoral (output and production costs), household (income and consumer demand), regional (households belonging and migrating between different regions) and aggregate (GDP, employment, trade). Important features of the DEMETRA model are:

• A disaggregation of economic activities into individual production processes
• A small open-economy assumption whereby domestic price changes do not impact world prices
• A separation of marketed and subsistence commodities with a consistent accounting of home production for home consumption (HPHC) allowing for the study of semi-subsistence agriculture where production and consumption decisions are not separable.
• An assumption of perfect competition i.e. prices and quantities are not subject to market power on the supply or demand side.

The model is calibrated using the 2017 Social Accounting Matrix (SAM) for Kenya developed in Mainar Causapé et al. (2018). The SAM comprises annual economic transactions structured around 53 sectors (11 of which account for households as home producers), 55 marketed commodities (and 18 home commodities) and 22 household groups (see SAM structure in Supplementary Material Tables A2-A4). Household groups are disaggregated into income quintiles for Nairobi and Mombasa and by the rural and urban division and agro-ecological zones for the rest of the country. The SAM was updated to 2020 using the model dynamics calibrated with the reported economic growth rates for 2018–2019 and a pre-COVID-19 growth rate for 2020 projected at 6%.

As with other CGE models, the DEMETRA model computes changes to prices and quantities of the captured economic transactions in annual time steps. Therefore, the simulations account for changes in exogenous variables (e.g. factor productivity) using annualised values of the COVID-19 impacts. Nevertheless, the quantifications in this study reflect short-term adjustments to the economy where price endogeneity guides the demand changes and the substitution decisions on both the demand and the supply side.

The lockdown in Kenya was imposed in late March which normally is the busiest month in the calendar of farmers when they begin planting key crops such as potatoes, cabbages, onions, pumpkins, sweet potatoes, and tomatoes. Farmers also start planting early season maize, millet, sorghum, hyacinth beans, common beans, black-eyed peas and kale. Therefore, to account for the seasonal labour demand in agriculture and for the cropping decisions being taken before the lockdown restrictions, the labour^3^ and land use in cropping activities are immobile. A further improvement in this specification could be done similarly to Feuerbacher et al. (2020) where temporary and permanent labour demand are clearly separated in the production functions.

Coupling the CGE model's results with a FS&N microsimulation module allows the computing of food sufficiency and food adequacy impacts at the household level. This FS&N module requires three inputs: households' consumption patterns (in expenditure and quantities converted to energy) from the latest KIHBS, energy and nutritional contribution of each food items (per gram) from Ramos et al. (2020), and changes in consumption of each food item, which come from the CGE model simulations (see FS&N module food categories and households characteristics in the Supplementary Material Tables A11 and A12).

Measuring the impact of food consumption at the household level has two relevant dimensions – food sufficiency and food adequacy. Food sufficiency concerns the change in kilocalories intakes per capita per day in a household. Given the composition of food consumption baskets of households (quantities) and using the food nutritional information of each food item (per gram) it is possible to compute the kilocalories consumed per capita per day in each household. When food market conditions change, due to COVID-19 or to a government measure, households also adapt and change the composition of their food consumption basket. These changes could increase or reduce the initial diet energy consumption (DEC) per day in each household. Non-parametric regressions of DEC changes provide the distribution of food sufficiency impacts across economic and nutritional characteristics of households.

The second food consumption metric concerns the food adequacy dimension, measured through the decomposition of calorie intakes by macronutrient. Each food item contributes to the average human nutrients' requirements differently. Focusing on the change in macronutrients intakes (MAC for proteins, fats, and carbohydrates), the change in the composition of food consumption baskets at the household level can improve (or deteriorate) the status of the current food adequacy. The considered macronutrients’ thresholds^4^ are those recommended by FAO (WHO, 2003).

### 3.2 COVID-19 scenarios

To capture the uncertainty around the evolution of the pandemic inside Kenya and abroad, the study includes three lockdown sets of assumptions:

• **V–V:** comprising the lockdown from April–June 2020 both in Kenya and abroad. In Kenya this lockdown implied restrictions over 10–11 weeks with some variations across economic sectors. The government fiscal and spending measures currently implemented were adopted as a response to this first wave of restrictions in Kenya and abroad.
• **V–W:** implying a single lockdown in Kenya but a second set of measures abroad imposing a new lockdown globally during the second part of 2020. This scenario together with the previous scenario V–V serve as reference since no additional strict measures have been officialy adopted in Kenya throughout 2020 while lockdown measures abroad have tended to vary from one region to another.
• **W–W:** comprising additional restrictions in Kenya in the last months of 2020 due to the second wave of cases in the country. The scale of these is assumed to have an effect 25% lower than the April–June measures to reflect some lessons learned from the first lockdown. At the time of writing, these assumptions remain hypothetical since the government responses to the new surge in cases in Kenya have so far implied curfews, limitations on public gatherings and a partial opening of the education and hospitality sectors.^5^ Therefore, the restrictions included here represent a worst case scenario for 2020, but also one that could develop at the beginning of 2021.

The COVID-19 lockdown impacts included in this analysis are differentiated across five channels: a decrease in labour productivity (to reflect the reduction in working hours), a reduction in exports and foreign tourism, a decrease in remittances, a shift of internal demand away from transport and hospitality services, and an increase in internal trade margins (to reflect disruptions to the food and other commodity distribution) – see Supplementary Material for more details.^6^ Due to limited evidence, the modelled shocks do not include any changes in business behaviour due to the uncertainty induced by the pandemic^7^ (e.g. investment decisions being postponed).

The Kenyan government response towards a short-term recovery from the COVID-19 impacts is modelled across several fiscal and public spending measures:

• Fiscal measures implemented through the Tax Law (Amendment) Act, 2020 effective April 1, 2020 (see Table A1 in Supplementary Material)
• The government spending to cover additional healthcare costs and to support the economic recovery as announced in the Economic Stimulus Plan and the COVID Spending Plan (see Table A7 in Supplementary Material). The total spending included in the analysis is KSh 39.3 billion (USD 360 million) including KSh 10 billion in direct transfers to support a fall in household income of vulnerable households. Given the sizeable amount of cash transfers, we further analyse their effectiveness for improving foods security impacts by including in the Supplementary Material a sensitivity analysis of total cash payments to households.

The policy scenarios also include additional government foreign loans and grants of USD 1.33 billion to partially cover the deficit resulting from the increase in government spending and the reduction in government revenues through lower tax levels. This amount corresponds to support packages from international donors to address the COVID-19 impacts. The rest of the public deficit resulting from the implementation of the government measures are funded through internal borrowing. At the same time, since the analysis of the impacts is limited to 2020, results do not account for the increase in debt services payments in the subsequent years resulting from pandemic-related public borrowing.

An overview of simulations covering both the different impact channels and the recovery measures is presented in Table 1. We combine impact channels under the All Impacts (V–V) simulation to show the compounded effects of the April–June lockdown in Kenya for the year 2020. The implemented government measures are added to the economy-wide model to show recovery after the lockdown resulting from these measures (All Measures V–V) but also to show their effectiveness under a potential second lockdown abroad (All Measures V–W) and in Kenya (All Measures W–W). The economy-wide results of this study are presented as deviations from the 2020 baseline which accounts for pre-COVID-19 projections of economic growth but excludes other shocks such as the floods and the locust invasion that have affected several countries in Eastern Africa in 2020. While it would have been desirable to integrate these in the baseline, the existing evidence for the quantification of their impacts remains limited.

**Table 1:** **-**List of simulations of COVID-19 impacts and government measures.

Simulations	Labour productivity	Export demand reduction	Internal demand changes	Increase in internal trade costs	Decrease in remittances	VAT reduction	Income tax reduction	Turnover tax reduction	Corporate tax reduction	Government spending	Foreign loans
**All Impacts (V–V)**	1x	1x	1x	1x	-23%	-	-	-	-	-	-
**All Measures (V–V)**	**1 x**	**1 x**	**1 x**	**1 x**	-23%	16% → 14%	-16%	3% → 1%	-16%	KSh 39.3 bn	USD 1.33 bn
**All Measures (V–W)**	**1 x**	**1.75x**	**1 x**	**1 x**	**-35%**	16% → 14%	-16%	3% → 1%	-16%	KSh 39.3 bn	USD 1.33 bn
**All Measures (W–W)**	**1.75x**	**1.75x**	**1.75x**	**1.75x**	**-35%**	16% → 14%	-16%	3% → 1%	-16%	KSh 39.3 bn	USD 1.33 bn

## 4 Results

### 4.1 Economy-wide impacts

**Fig. 3:**
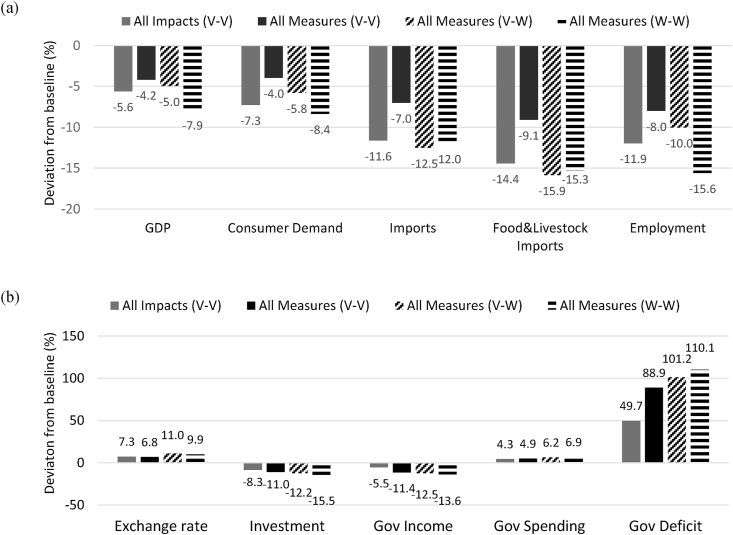
**Macroeconomic impacts in 2020 across lockdown scenarios.***Results are expressed as deviations from pre-COVID-19 baseline values for 2020. V–V represents the reference values for the April-June 2020 lockdown, V–W implies a second lockdown occurring only outside Kenya, while in W–W both Kenya and rest of the world adopt additional restrictive measures in the second part of 2020.*

The simulation results show that the April–June 2020 lockdown in Kenya could have led to a 5.6% reduction in GDP relative to the baseline (Fig. 3a) with the largest contributor being the drops in labour productivity (-4% GDP impacts), export demand (-0.8%) and internal demand (-0.4%) – see individual impact channel effects in Supplementary Material Figure A1. With all impacts combined, consumer demand would have reduced by -7.3% while general employment would have dropped by 11.9% in annual terms. The reduction in aggregate exports demand by 8.1% together with the decrease in remittances would have led to a depreciation of the Kenya Shilling (an increase in the exchange rate – Fig. 3b) and a consequent price increase of imported commodities. Therefore, under higher prices, aggregate imports and food imports would have declined by 11.6% and 14.4% respectively.

With all government measures included, the GDP impacts of the April–June lockdown decrease to -4.2%, total supply to -4.1%, consumer demand to -4.0% and imports to –7%. Therefore, these measures lead to a short-term recovery as the tax rate reductions, cash transfers and increases in public spending determine an increase in real income, a boost to internal demand and a partial recovery of the economic sectors. The food (food crops, livestock and processed food) and healthcare sectors benefit the most from these measures (see additional results in Supplementary Material Figures A2). Nevertheless, the fiscal relaxation and the increased public spending also widen the differences between tax revenues and total government spending and lead to a steep public deficit increase.

The simulations of a potential second lockdown occurring towards the end of 2020 show a further substantial impact on the macroeconomic indicators. A new lockdown taking place only outside Kenya (V–W scenario) determines a further 0.8% GDP reduction while imports decline to -12.5% relative to baseline values (food and livestock imports by -15.9%). With a second wave of restrictions extended to Kenya (W–W scenario), GDP contracts by 7.9% and consumer demand drops by 8.4% relative to the baseline values. Scenarios V–W and W–W indicate that while the external factors have a visible effect on GDP performance, the largest impacts come from the internal channel of labour productivity. However, they also illustrate the exposure of food supply, through imports, to the changes in the trade balance when foreign demand for Kenyan exports reduces.

**Fig. 4:**
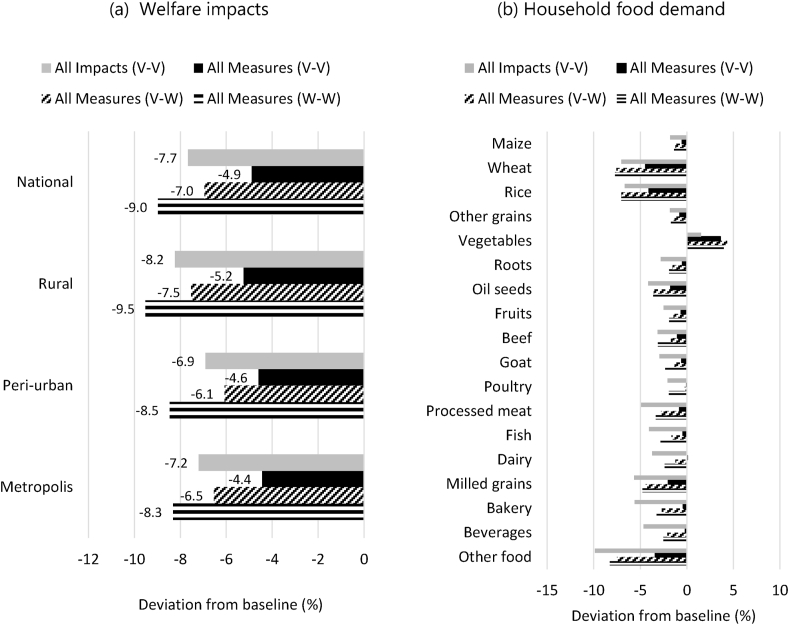
**Impacts on household welfare and aggregate food demand.***Welfare changes are calculated as income changes deflated by the consumer basket price changes of each household group in the model.*

The combined impacts of the April–June lockdown would have led to a 7.7% decrease in welfare^8^ across Kenyan households, which is mainly driven by the fall of the their income. Rural households are the hardest affected with a welfare decline of 8.2% (Fig. 4a) followed by those in metropolitan areas (Nairobi and Mombasa) facing a 7.2% welfare decline. The government measures are supportive of a partial welfare recovery. The largest effects occur in the metropolitan areas and rural areas which rebound from the COVID-19 impacts by 2.8 and 3 percentage points respectively. At the same time, the absolute reduction in rural welfare remains the largest across the three household groupings. Under a second wave of restrictions in Kenya, these income recovery trends would, however, be more than offset with rural households welfare declining by 9.5% relative to baseline values for 2020. Revenue from labour is affected as employment across economic sectors declines. The largest reductions are obtained for the low and semi-skilled workforce. Furthermore, the government measures appear as more effective for high-skilled jobs compared to the other categories. These employment dynamics have thus the largest negative consequences over households for which low-skilled work represents the largest source of revenue.

With the lower income resulting from the lockdown impacts, household demand for food commodities would generally decrease (All Impacts V–V in Fig. 4b), in spite of a shift in household spending determining a larger share of total income dedicated to food. The demand change would mostly be felt for market commodities, as household would continue to rely on home production by similar levels as in the baseline (see Figure A4 in Supplementary Material). Vegetables and poultry are the only categories for which demand increases as these also face some of the largest price reductions due to the contraction of a large demand driver - tourism.

The government measures (All Measures V–V) determine a partial recovery of food demand as consumption losses resulting from the lockdown are reduced by more than half for most food commodities. Wheat, rice and oil seeds are the categories with the lowest effect from the recovery interventions, nevertheless the effect is still sizeable. A number of food categories (meat products, dairy, fish, roots, fruits, bakery and beverages) have a net increase in demand relative to the baseline indicating a change in the composition of consumption baskets. However, a second lockdown abroad (All Measures V–W) poses challenges for demand across almost all food categories as the depreciation of the Kenyan Shilling renders imported goods more expensive. Additional restrictions inside Kenya (All Measures W–W) leads to a further reduction in food demand due to income effects.

### 4.2 Food security and nutrition impacts

Results of the FS&N microsimulation indicate that, at the national level, DEC per capita declines across all scenarios. The worst food consumption impact is obtained in absence of government measures (All Impacts V–V) – Table 2. Nationally, 3.32 percentage points of households fall below the daily threshold of 2,250 kilocalories per capita. With the income supporting measures by the government this value decreases to 1.26 percentage points (All Measures V–V). Among the three regional household groupings, rural households see the highest reduction in DEC/capita. These are also the households with the lowest DEC per capita in the pre-COVID-19 baseline (see Table A12 in Supplementary Material). Therefore, government's measures, while effective in lowering DEC impacts at an aggregate level, favour urban households. Another point of concern is that households where child stunting is present (Min HAZ <= - 2) see a further decrease in calorie intake and do not recover to baseline values after the government intervention, even when the shift in internal demand lead to a positive but small effect for these households (Table A8 in Supplementary Material).

**Table 2:** **Prevalence of food sufficiency and of unbalanced diets across households (% of all households).***The threshold for food sufficiency is a daily calorific requirement of 2,250 kilocalories. An unbalanced diet implies not meeting at least one of the WHO macronutrient intake proportions.*

HH	Baseline	All Impacts (V–V)	All Measures (V–V)	All Measures (V–W)	All Measures (W–W)
*Food sufficiency (DEC* per capita *per day)*					
**National**	**39.04**	**35.72**	**37.78**	**36.62**	**36.30**
Metropolis	40.08	38.38	40.48	39.58	39.68
Peri-urban regions	41.01	37.64	39.64	38.51	38.11
Rural regions	37.81	34.40	36.48	35.29	34.98
Min HAZ <= -2	20.94	18.41	20.13	18.97	18.97
*Prevalence of an unbalanced diet – WHO thresholds*					
**National**	**2.73**	**4.41**	**4.40**	**4.40**	**4.40**
Metropolis	6.48	6.38	6.38	6.38	6.38
Peri-urban regions	3.60	5.10	5.08	5.06	5.08
Rural regions	1.93	3.86	3.86	3.87	3.85
Min HAZ <= -2	1.07	3.09	3.05	3.09	3.09

Under a second wave of restrictions abroad (All Measures V–W), rural households appear as the most affected in their caloric intakes due to the negative impact in almost all food items except for vegetables. At the national level, the second lockdown abroad impacts in particular the consumption of cereals, bread, oils and fats which are very important for their caloric content in the diet of Kenyan households (see Table A11 in Supplementary Material).

Combined with a reduction in DEC, dietary balance is also negatively affected as the percentage of households not meeting at least one of the WHO macronutrient intake proportions grows from 2.7% to 4.4%. Nationally, government measures marginally improve dietary balance outcomes only for peri-urban households, while increase in the aggregate prevalence of an unbalanced diet is largely driven by rural households. This deterioration in food adequacy is determined by changes in the food consumption composition where at least one of the three macronutrients (proteins, fats and carbohydrates) is consumed excessively relative to the others (see Table A10 in Supplementary Material). Households with child stunting are also among the most impacted with the incidence of an unbalanced diet increasing from 1% to 3%.

**Fig. 5:**
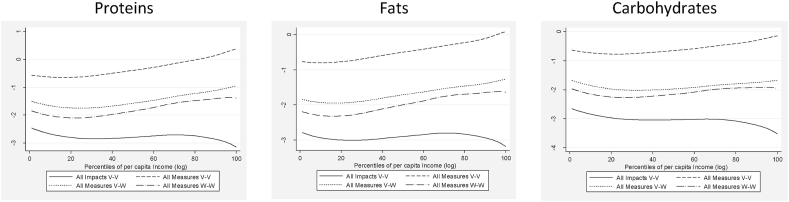
Macronutrient intake changes across per capita income percentiles.

Analysing the macronutrient intakes across income per capita percentiles (Fig. 5), the lockdown leads to a more pronounced reduction for households in the middle-income percentiles and those in the top percentiles (All Impacts V–V). Through the government economic measures, the impact curves are reversed with a sizeable recovery from around -3% to less than -1% for all three macronutrients. However, the recovery is stronger for households with a higher income per capita, notably on the fat and protein intakes.

## 5 Discussion and conclusions

The right to adequate food of acceptable quality and freedom from hunger for all Kenyans is enshrined in the Kenya's 2010 Constitution. Food and nutrition insecurity in the country is closely related to poverty with the chronically food insecure population also suffering from extreme poverty (GoK, 2017). Achieving national food security and improving nutrition by year 2022 are key government objectives, being prioritized in Kenya's Vision 2030 and in the government's Big Four Action Plan.^9^ The proportion of food poor individuals declined over the years from 45.8% in 2006 to 32.0% in 2016 and the food poor individuals decreased from 16.3 million in 2006 to 14.5 million in 2016 (KNBS, 2018).

Nonetheless, this study indicates that the economic impacts of the COVID-19 lockdown may reverse the food security improvements from the past years. The April–June lockdown might have determined a significant decrease in GDP and household income which have then translated into a lower demand for food commodities. The government fiscal and public spending measures have partially alleviated the negative welfare impacts with direct implications for food security. However, the results show an uneven recovery across household groupings as rural households and those with child stunting see the lowest improvement in calorie intake and macronutrient balance. With government measures, metropolitan households (Nairobi and Mombasa) face the lowest income and food consumption impacts, followed by those in peri-urban areas.

These findings make the case for additional efforts through food and income support programmes aimed at the most vulnerable population. According to World Bank (2020c) and MoLSP (2020), only a small fraction of Kenyan households has received some form of safety net assistance while many continue to skip meals as a result of the pandemic (World Bank, 2020d). One important avenue of intervention to further improve short-term food security outcomes is to scale up the cash payments program and to extend the beneficiary base to the bottom 40% income percentiles. This represents the population preponderantly living in rural areas which was facing low calorie intakes even before the pandemic and for which income and food consumption recovery lag behind. The sensitivity analysis regarding the size of these transfers shows nevertheless a trade-off between economic output recovery and food security of lower income households if cash payments are not backed up by further international assistance.

Exploring the impacts of a potential second wave of restrictions in the country, we obtained a diminished food security recovery under the existing government measures. Furthermore, the food security status in Kenya is significantly dependent on the evolution of the pandemic outside the country. The additional negative impact of lower exports on the trade balance and on the domestic exchange reduces the affordability of imported food commodities and leads to a lower consumption of cereals, bread, oils and fats. At the same time, under a renewed set of restrictions in Kenya and abroad, the capacity of the government to address additional economic constraints may be limited given that the first set of measures has already significantly expanded public deficit. The increase in foreign debt could also make the contracting of new loans more difficult in the short run unless the international COVID-19 assistance of USD 1.33 billion will be scaled up and debt would be restructured in a way that medium-term recovery is not overburdened by debt service repayments.

The authors acknowledge the limitations in using a general equilibrium framework in countries such as those in Sub-Saharan Africa where market failures are present. Also, the food consumption composition at the onset of the pandemic may have evolved from the one captured in the latest KIHBS from 2015/2016. Nevertheless, the income and food security impacts across the scenarios offer an ordinal picture on the most important impact channels (productivity declines due to mobility restrictions, reduced exports and remittances) also reported for Kenya in Kansiime et al. (2021). While it is still early to find evidence on the actual impacts of the second wave of COVID-19 cases in Kenya which occurred in the last months of 2020, the data available up to October 2020 (KNBS, 2020b) confirms a gradual recovery in economic activity after the first lockdown but also a similar depreciation of the Kenyan Shilling and a contraction of food imports of a similar size as those in this study. Also, the GDP results obtained here for the April–June lockdown (All Measures V–V scenario) are in line with the -4.3% projected GDP deviation in (World Bank, 2020e).

The food security findings confirm that the pandemic may generate additional undernourished people worldwide as suggested by preliminary projections by the UN Food and Agriculture Organisation who estimated a possible increase between 83 and 132 million people (FAO, IFAD, UNICEF, WFP, WHO, 2020). Adequate policy measures by local governments, duly supported financially by the international community, could limit the negative effects on global food security. When designing these measures, government should target these interventions towards the more fragile sectors of the population to allow for an equitable recovery from the pandemic.

## Declaration of competing interest

The authors declare that they have no known competing financial interests or personal relationships that could have appeared to influence the work reported in this paper.

## Footnotes

1 For instance, as opposed to results in Laborde et al. (2020b), Kenya's workforce mobility between rural and urban environments has proved to be limited during and after the lockdown period – see (KNBS, 2020a, p. 22, p. 22).

2 DEMETRA is a development of the STAGE_DEV models documented in Aragie et al. (2017). STAGE_DEV is a variant of STAGE_2 (McDonald, 2015) that incorporates a series of additional behavioural relationships that better account for economic relationships in developing countries, such as the dual role of semi-subsistent agricultural households, a nested consumption function, the endogeneity of the functional distribution of income, domestic migration and factor market segmentation. The recursive dynamic version of DEMETRA is derived from the STAGE_DYN model and STAGE_DEV_DYN models.

3 The model accounts for three labour types (skilled, semi-skilled and low-skilled). The model closure fixes the baseline low-skilled labour allocation in both market and subsistence crop production.

4 The ranges of population nutrient intake goals for energy-supplying macronutrients are expressed as percentage of total energy: fat (15–30%), carbohydrate (55–75%) and protein (10–15%).

6 In the Supplementary Material, Table A5 presents labour productivity decreases by sector and Table A6 presents changes in export levels for the V–V set of assumptions.

7 As a recursive dynamic CGE model, agent behaviour in the DEMETRA model is not forward-looking.

8 Calculated using the Equivalent Variation (EV) welfare measure. The welfare impacts are thus determined as EV scenario values for each household group relative to the EV values in the pre-COVID-19 baseline for 2020.

9 See https://www.president.go.ke/also Republic of Kenya, 2018 Budget Policy Statement pp 22-24.

## Footnotes

5 https://www.president.go.ke/2020/11/04/the-thirteenth-13th-presidential-address-on-the-coronavirus-pandemic-wednesday-4th-november-2020-at-state-house-nairobi/.
