# Supplementary material for: Food security and welfare changes under COVID-19 in Sub-Saharan Africa: Impacts and responses in Kenya
Source: Glob Food Sec. 2021 Mar;28:100514. doi: 10.1016/j.gfs.2021.100514 (PMC7938699; doi:10.1016/j.gfs.2021.100514)
Supplement: Multimedia component 1 [file mmc1.pdf]

Food security and welfare changes under COVID-19: impacts and short-term responses in Kenya

Victor Nechifor, Maria Priscila Ramos, Emanuele Ferrari, Joshua Laichena, Evelyne Kihiu, Daniel Omanyo, Rodgers Musamali, Benson Kiriga

Supplementary Material

Impact channel definition

(1) Labour productivity: sector-specific changes to monthly labour productivity due to social distancing protocols in the range of -10% and -83% (see Table A5). The productivity calculations were based on the reduction of worked hours in the different economic sectors in Kenya as resulting from the May 2020 household survey (KNBS, 2020). These monthly estimates were multiplied by the lockdown duration assumptions in the three scenarios. For the scenarios comprising a single period of restrictions in Kenya (V-V and V-W) the annualised productivity changes were calculated based on a duration of restrictions of 10-11 weeks (depending on the sector), while outside the lockdown period the productivity was considered to return to normal levels. With a second set of restrictions in Kenya, a further reduction in productivity was added of the same 10-11 week duration in the last months of 2020 of a magnitude 25% lower to reflect some lessons learned from the first lockdown. The productivity shocks were applied in each sector affected with the same sector-specific productivity value for all three labour types in the model (high skilled, semi-skilled, low-skilled).

(2) Internal trade: annualized internal trade margins were increased by 1% in the V-V scenario and 2% in the V-W and W-W scenarios to reflect the higher cost of transportation and storage during the lockdown months – the KNBS April 2020 Survey reflected a 5% increase in transport cost for the month of April 2020 (KNBS, 2020). This additional cost increase was applied over the duration of mobility restrictions (one fifth of the year for the April-June lockdown period), while trade costs outside the lockdown period were considered to return to normal levels.

(3) Export and tourism demand: changes in export volumes for specific commodities during the lockdown months with values for the April-June wave obtained from MoALFC , KEPSA's findings on the impact of COVID-19 on businesses (KEPSA, 2020a, 2020b, 2020c) and the June 2020 National Food Balance Sheet (MoALFC, 2020). For commodity groups where data was unavailable (notably industry), a reduction of 5.2% was included to reflect the average export decreases obtained for Sub-Saharan Africa in the global study by the World Trade Organization (WTO, 2020). Export volumes after the April-June lockdown were assumed to gradually return to normal values towards the end of 2020. The annualised reduction values are included in the Supplementary material (Table A6).

(4) Internal demand: changes in household demand for specific commodity groups: a) A decrease of household budget allocation to transportation by -15% (equivalent of a 75% reduction in transport demand during 2.5 months) and to hospitality by -54% (the same reduction as for foreign tourism demand) b) An increase in household budgets for all other commodities to

compensate for this reduced spending on transportation and hospitality. In the DEMETRA model, this was implemented by recalibrating the technical shares of the individual consumer commodity groups entering the household demand functions.

(5) Remittances: a reduction in remittances to households by 23% from baseline values – this reduction is in line with remittance reduction for Sub-Saharan Africa in World Bank (2020b).

2015/16 Kenya Integrated Household Budget Survey – summary

Based on data from the KHIBS 2015/2016, the national average of daily DEC per capita is 1,970 kcal, which is quite similar across regions (but rural with a greater dispersion). Moreover, 46% of households at National level have a balanced diet in terms of the three macronutrients recommended goal for energy-supplying macronutrients (i.e., fats between 10-15%, proteins between 15-30% and carbohydrate between 55-75% of calories daily consumed) while 2.7% of them does not meet any of these goals. Unbalanced diet does not mean necessarily being below the minimum bound but also to be above the maximum threshold (Ramos et al., 2020).

Kenyan households (National level) spend on average more a than 50% of their budget in food and display a quite diversified diet consuming 11 of 16 food groups accounted for the HCE-DDS. However, disparities rise across regions. For instance, in rural areas households spend on average 65% of their income in food and a lower HCE-DDS (10), while in Nairobi and Mombasa (Metropolis) households spend 40% of their budgets in food and display a best diversified diet (HCE-DDS of 12) (Ramos et al., 2020).

Additional information

Table A1 - Tax changes in response to COVID-19 economic impacts

Tax	Change	Implementation in the DEMETRA model
VAT	Reduction from 16% to 14%	A 12.5% reduction in the model sales tax rate.
Income tax	100 % tax relief for persons earning gross monthly income of up to KSh 24,000.<br><br>Reduction of resident Personal Income Tax Rate (Pay-As-You-Earn) top rate from 30% to 25%.	Due to data limitations on the distribution of households across tax brackets, in the current version of study the income tax was reduced by 16.6% across all household groups represented in the model. This corresponds to the effective reduction of personal income tax obtained for the top rate from 30% to 25%
Turnover tax	Reduction from 3% to 1% with taxable turnover thresholds increased from an income of between KSh 1 million (US$ 10,000) to KSh 50 million (US$ 500,000) for MSMSEs.	A two-thirds reduction in the model indirect tax rate.
Corporate tax	Reduction in corporate tax from 30% to 25% for residents and increase; withholding tax rate on dividend payable to non-residents from 10% to 15%.	A 16.6% reduction in enterprise direct tax rate of the model.

Table A2 - Economic sectors in the Kenya 2017 Social Accounting Matrix

**Economic sectors**		
Food crops	Grain milling	Electricity, gas and steam
Cotton and fibres	Bakery	Construction
Sugar cane	Beverages	Wholesale and retail trade
Coffee	Other food processing	Accommodation and food services
Tea	Textiles	Transportation and storage
Tobacco	Other chemicals	Information and communication
Flowers and other crops	Fertilizers - N	Finance and insurance
Livestock	Fertilizers - P	Real estate
Dairy	Fertilizers - K	Other services
Fishing	Non-metal minerals	Public administration
Forestry	Machinery and other equipment	Health and social work
Minerals	Other manufacturing	
Meat processing	Water supply and sewage	

Table A3 - Marketed commodities in the Kenya 2017 Social Accounting Matrix

**Commodities**		
Maize	Fishing	Other manufacturing
Wheat and barley	Forestry	Water supply and sewage
Rice	Minerals	Electricity, gas and steam
Other grains	Other meat	Construction
Other roots	Milled grains	Wholesale and retail trade
Other oilseeds	Bakery	Electricity, gas and steam
Fruits and nuts	Goat	Road infrastructure
Vegetables	Other food	Irrigation infrastructure
Cotton	Leather and footwear	Other infrastructure
Sugars	Minerals	Construction other
Coffee	Paper and printing	Wholesale and retail trade
Tea	Petroleum products	Accommodation and food services
Tobacco	Other chemicals	Transportation and storage
Flowers and other crops	Fertilizers - N	Information and communication
Beef	Fertilizers - P	Finance and insurance
Dairy	Fertilizers - K	Real estate
Poultry	Non-metallic minerals	Other services
Tea	Machinery and other equipment	Public administration
Other livestock	Other manufacturing	Health and social work
Textiles	Water supply and sewage	Education

Table A4 - Household grouping in the Kenya 2017 Social Accounting Matrix

**Household groups**		
High Rainfall – Rural	Nairobi - Quintile 1	Mombasa - Quintile 1
High Rainfall – Urban	Nairobi - Quintile 2	Mombasa - Quintile 2
Semi-Arid North - Rural	Nairobi - Quintile 3	Mombasa - Quintile 3
Semi-Arid North - Urban	Nairobi - Quintile 4	Mombasa - Quintile 4
Semi-Arid South - Rural	Nairobi - Quintile 5	Mombasa - Quintile 5
Semi-Arid South - Urban		
Coast - Rural		
Coast – Urban		
Arid North - Rural		
Arid North - Urban		
Arid South - Rural		
Arid South - Urban		

Table A5 - Changes to labor productivity across sectors

**Sector**	**Lockdown duration (weeks)**	**Monthly productivity loss**	**Annualised productivity loss (V-V and V-W scenarios)**	**Sector**	**Lockdown duration (weeks)**	**Monthly productivity loss**	**Annualised productivity loss (V-V and V-W scenarios)**
Food crops	10	-17%	-3.2%	Petroleum products	10	-25%	-4.8%
Cotton and fibres	10	-17%	-3.2%	Other chemicals	10	-25%	-4.8%
Sugar cane	10	-17%	-3.2%	Fertilizers - N	10	-25%	-4.8%
Coffee	10	-17%	-3.2%	Fertilizers - P	10	-25%	-4.8%
Tea	10	-17%	-3.2%	Fertilizers - K	10	-25%	-4.8%
Tobacco	10	-17%	-3.2%	Non-metal minerals	10	-13%	-2.4%
Flowers and other crops	10	-17%	-3.2%	Machinery and other equipment	10	-25%	-4.8%
Livestock	10	-17%	-3.2%	Other manufacturing	10	-25%	-4.8%
Dairy	10	-17%	-3.2%	Water supply and sewage	10	-21%	-4.0%
Fishing	10	-17%	-3.2%	Electricity, gas and steam	10	-10%	-2.0%
Forestry	10	-13%	-2.4%	Construction	10	-48%	-9.2%
Minerals	10	-13%	-2.4%	Wholesale and retail trade	10	-27%	-5.2%
Meat processing	10	-17%	-3.2%	Accommodation and food services	11	-63%	-13.0%
Grain milling	10	-17%	-3.2%	Transportation and storage	11	-25%	-5.2%
Bakery	10	-17%	-3.2%	Information and communication	10	-27%	-5.2%

Beverages	10	-17%	-3.2%	Finance and insurance	10	-25%	-4.8%
Other food processing	10	-17%	-3.2%	Real estate	10	-42%	-8.0%
Textiles	10	-25%	-4.8%	Other services	10	-50%	-9.6%
Leather and footwear	10	-25%	-4.8%	Public administration	11	-4%	-0.9%
Wood products	10	-25%	-4.8%	Health and social work	11	-10%	-2.2%
Paper and printing	10	-25%	-4.8%	Education	11	-83%	-17.4%

Table A6 - Changes to commodity exports

**Commodity**	**Annualised exports reduction**	**Commodity**	**Annualised exports reduction**
Maize	-45.8%	Forestry	-5.2%
Wheat and barley	-45.8%	Minerals	-5.2%
Rice	-45.8%	Other meat	-5.2%
Other grains	-34.4%	Milled grains	-5.2%
Other roots	-34.4%	Bakery	-5.2%
Other oilseeds	-5.2%	Goat	-5.2%
Fruits and nuts	-34.4%	Other food	-5.2%
Vegetables	-34.4%	Textiles	-5.2%
Cotton	-5.2%	Leather and footwear	-5.2%
Sugars	-5.2%	Minerals	-5.2%
Coffee	-5.2%	Petroleum products	-5.2%
Tea	-2.7%	Other chemicals	-5.2%
Tobacco	-5.2%	Non-metallic minerals	-5.2%
Flowers and other crops	-22.5%	Machinery and other equipment	-5.2%
Beef	-5.2%	Other manufacturing	-5.2%
Dairy	-5.2%	Wholesale and retail trade	-5.2%
Poultry	-5.2%	Accommodation and food services	-45.8%
Goat	-5.2%	Transportation and storage	-5.2%
Other livestock	-5.2%	Finance and insurance	-5.2%
Fishing	-5.2%		

Table A7 - Additional government expenditure measures included in the analysis (in million KSh)

Area	Category	Amount***
	Total	*6,470*
	Roads, foot bridges etc.	5,000
	Rehabilitation of wells, water pans and underground tanks	850
Infrastructure	Drilling of Boreholes in Informal Settlements in Nairobi	620
Health	*Total*	*11,940*
	Recruitment of health workers	1,000
	COVID related health response measures	6,240
	COVID expenditures on health	3,000
	Additional 5000 healthcare workers	1,700
Education	*Total*	*6,500*
Tourism	*Total*	*4,000*
	Soft loans to hotels and related establishments	2,000
	Engagement of 5,500 community scouts	1,000
	160 community conservancies	1,000
Public administration	*Total*	*400*
Cash transfers to vulnerable groups	*Total*	*10,000**
Total spending included		*39,310***

* For Nairobi and Mombasa, transfers where distributed across the lower 3 income quintiles with the following shares: 10% to quintile 3, 25% to quintile 4 and 65% to quintile 5. Outside Nairobi and Mombasa, household groups in the economic model are not disaggregated across income groups; total transfers were allocated proportionally based on the pre-COVID-19 government transfer patterns in the Social Accounting Matrix.

** Other stimulus spending such as liquidity support in the form of loans to banks and enterprises, early VAT refunds and government pending bill payments were not included in the analysis since these measures could not be captured by the modelling framework.

*** The increase in government expenditure on goods and services was implemented in the DEMETRA model by increasing the government demand for the corresponding commodities in the SAM at pre-COVID-19 prices for 2020.

Additional results

Figure A1 - Macroeconomic impacts of the April-June 2020 COVID-19 lockdown by impact channel

(a)

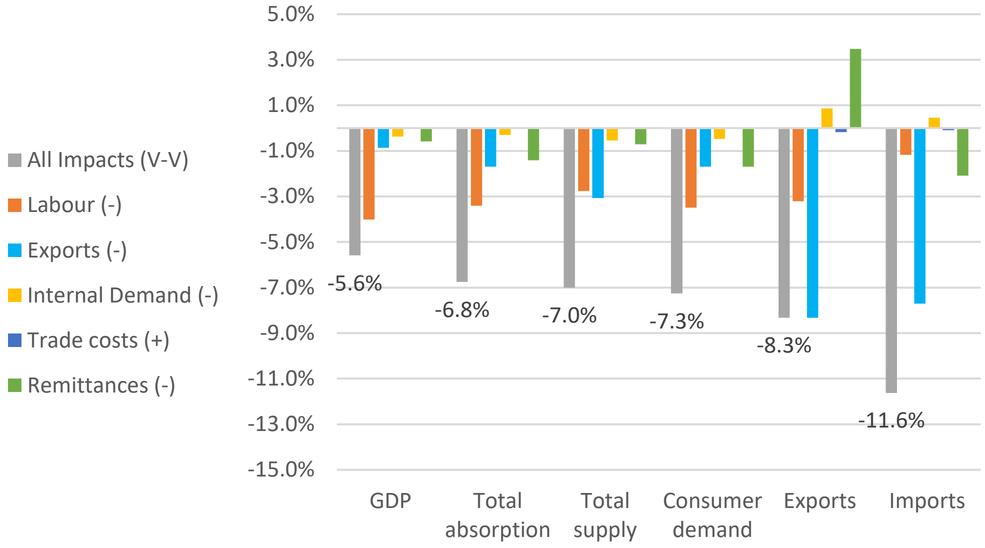

(b)

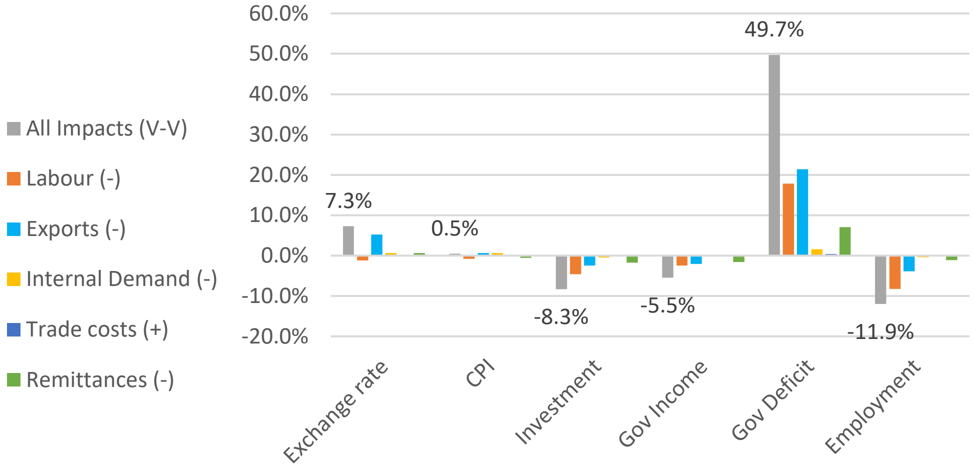

Figure A2 - Output by sector - changes relative to the 2020 baseline

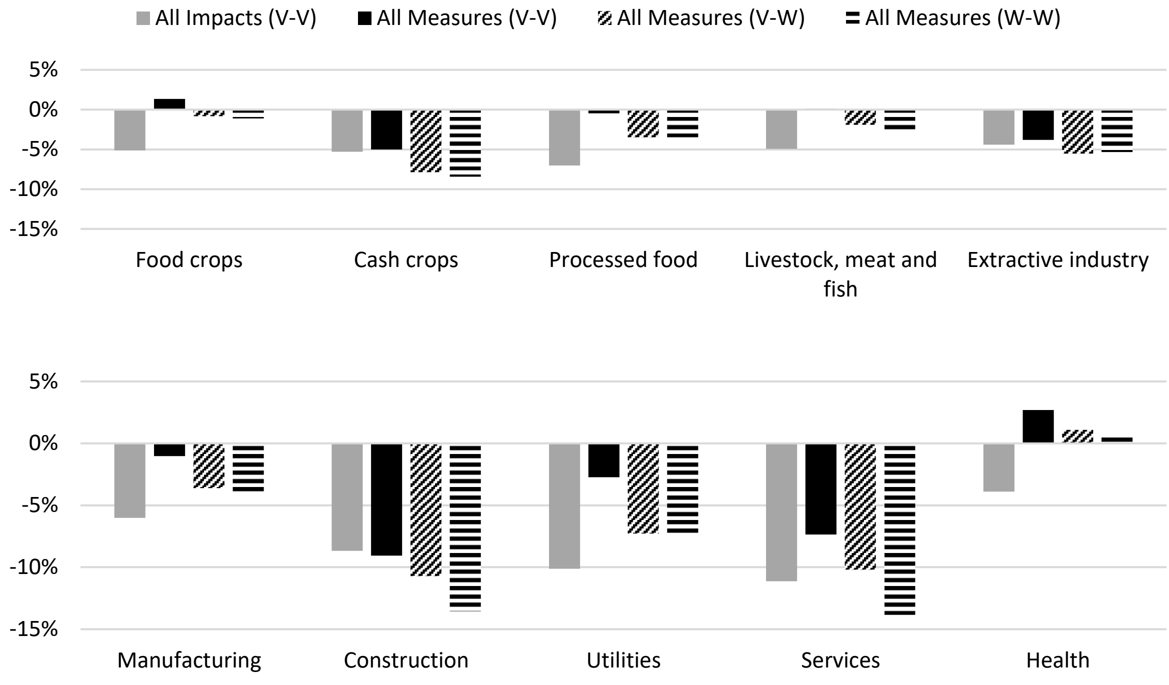

Figure A3 – Employment changes by skill level

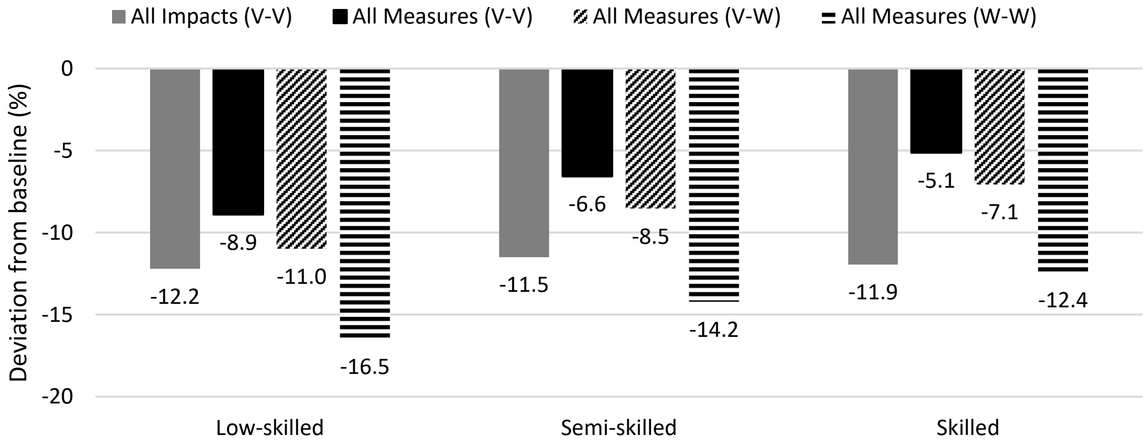

Figure A4 - Household demand of food commodities by home production and market sources (in billion KSh at fixed prices)

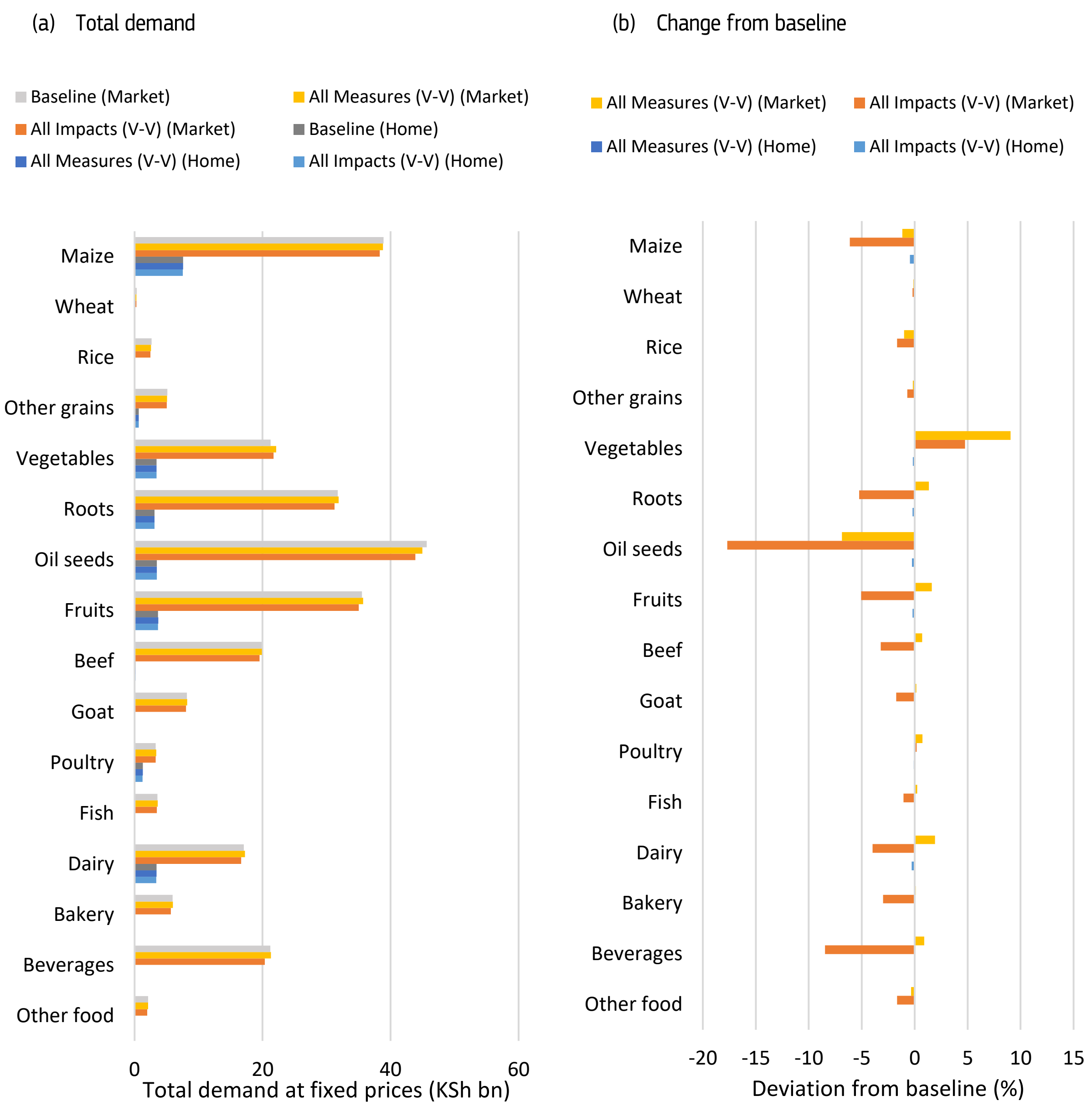

Table A8 - Prevalence of food sufficiency and of unbalanced diets across households. Decomposition of the All Impacts (V-V) scenario by impact channel. The threshold for food sufficiency is a daily calorific requirement of 2,250 kilocalories. An unbalanced diet implies not meeting at least one of the WHO macronutrient intake proportions. Number of HH based on the KIHBS 2015/2016 sample.

HH	Baseline	All Impacts (V-V)	Labour	Trade Costs	Export Demand	Internal Demand	Remittances
Food sufficiency (DEC per capita per day)							
**National**	**39.04**	**35.72**	**36.96**	**38.47**	**37.45**	**39.22**	**37.42**
Metropolis	40.08	38.38	38.98	40.08	40.08	41.48	38.38
Peri-urban regions	41.01	37.64	38.91	40.36	39.19	40.83	39.30
Rural regions	37.81	34.40	35.66	37.25	36.23	38.12	36.25
Min HAZ <= -2	20.94	18.41	19.53	20.60	19.87	21.29	19.83
Prevalence of an unbalanced diet – WHO thresholds							
**National**	**2.73**	**4.41**	**4.43**	**4.45**	**4.43**	**4.45**	**4.44**
Metropolis	6.48	6.38	6.48	6.48	6.48	6.48	6.38
Peri-urban regions	3.60	5.10	5.14	5.15	5.11	5.14	5.14
Rural regions	1.93	3.86	3.86	3.88	3.88	3.89	3.89
Min HAZ <= -2	1.07	3.09	3.00	3.00	3.05	3.00	3.00

Note: National sample is 21625 households; where 5% is from Metropolis, 35% from peri-urban regions and 60% from rural regions. According to this sample from the KIHBS 2015/2016 2233 households has at least 1 stunted child and this is the size of the sample represented in the household category “Min HAZ <=-2”.

Table A9 - Changes in DEC per capita per day across income deciles

Deciles Income per capita	Baseline (daily DEC pc)	All Impacts (V-V)	All Measures (V-V)	All Measures (V-W)	All Measures (W-W)
		% changes			
1	1160	-2.77	-0.69	-1.78	-2.09
2	1589	-3.00	-0.80	-1.99	-2.32
3	1828	-3.05	-0.81	-2.04	-2.35
4	2009	-3.07	-0.73	-2.01	-2.27
5	2208	-3.09	-0.70	-2.01	-2.25
6	2309	-2.87	-0.38	-1.71	-1.82
7	2505	-2.93	-0.50	-1.76	-1.99
8	2708	-3.01	-0.51	-1.82	-2.04
9	2980	-2.87	-0.29	-1.65	-1.70
10	3341	-3.29	-0.06	-1.49	-1.77

Table A10 - Proportion of the households within, below or above the ranges of macronutrient intake goals (FAO-WHO, 2003) across scenarios

	**Baseline**	**All Impacts (V-V)**	**All Measures (V-V)**	**All Measures (V-W)**	**All Measures (W-W)**
A balanced diet	46.07%	45.97%	45.77%	45.99%	45.91%
A diet that does not meet any of the three recommended goals for energy-supplying macronutrients	2.73%	4.41%	4.40%	4.40%	4.40%
Dietary energy provided by protein below the lower recommended threshold (10%)	29.38%	28.29%	28.49%	28.25%	28.34%
Dietary energy provided by fat below the lower recommended threshold (15%)	20.67%	20.04%	20.05%	19.98%	20.06%
Dietary energy provided by carbohydrate below the lower recommended threshold (55%)	5.85%	5.58%	5.57%	5.60%	5.55%
Dietary energy provided by protein above the upper recommended threshold (15%)	6.95%	8.54%	8.65%	8.64%	8.58%
Dietary energy provided by fat above the upper recommended threshold (30%)	9.98%	11.46%	11.42%	11.42%	11.42%
Dietary energy provided by carbohydrate above the upper recommended threshold (75%)	13.56%	14.94%	15.03%	14.98%	15.02%

Table A11 - Average kilocalories contribution (total and decomposed by macronutrient) of each type of food per 100 g

	**Kcal**	**FAT**	**PRO**	**CAR**
**Sectors**	*average kilocalories / 100g*			
Bakery	358	31	15	311
Beef	157	87	69	0
Beverages	225	13	51	161
Dairy	103	55	23	24
Fish	259	71	186	2
Fruits and nuts	81	32	4	46
Corn	324	36	30	259
Other meat	165	97	66	2
Other grains	272	30	30	213
Oils	592	453	46	93
Other food	75	16	11	48
Poultry	207	117	75	15
Rice	349	7	29	313
Roots	107	2	9	96
Vegetables	32	2	8	21
Wheat and barley	340	16	45	279

Source: own elaboration based on the Food Composition Table of Kenya 2018.

Table A12 - Average DEC per capita per day and macronutrient's decomposition (kcal.; shares in DEC) computed for Kenya regions/cities/quintiles.

			DEC	Fat	Protein	Carbohydrate
**Geo**	**City/AEZ**	**Quintile**	**kcal. per capita per day**	**kcal. and *share* in DEC**	**kcal. and *share* in DEC**	**kcal. and *share in* DEC**
Metropolis	Mombasa	Q1	1299.8	259.0	156.2	884.6
				0.20	*0.12*	*0.68*
		Q2	1638.4	359.8	194.4	1076.3
				0.22	*0.12*	*0.66*
		Q3	1793.0	422.5	219.7	1143.0
				0.24	*0.12*	*0.64*
		Q4	2265.7	569.6	277.5	1414.0
				0.25	*0.12*	*0.62*
		Q5	2647.8	654.9	329.6	1633.1
				0.25	*0.12*	*0.62*
	Nairobi	Q1	1504.7	347.0	160.3	997.0
				0.23	*0.11*	*0.66*
		Q2	2023.3	487.8	226.0	1308.2
				0.24	*0.11*	*0.65*
		Q3	2313.7	577.5	271.1	1460.5
				0.25	*0.12*	*0.63*
		Q4	2968.8	781.1	338.9	1836.8
				0.26	*0.11*	*0.62*
		Q5	3446.8	918.6	421.7	2021.0
				0.27	*0.12*	*0.59*
Peri-urban regions	Arid North		2224.1	470.9	230.1	1506.4
				0.21	*0.10*	*0.68*
	Arid South		2030.1	436.5	200.3	1392.6
				0.22	*0.10*	*0.69*
	Coast		2487.2	594.7	273.0	1607.4
				0.24	*0.11*	*0.65*
	High Rainfall		2240.5	516.2	258.4	1453.0
				0.23	*0.12*	*0.65*
	Semi-arid North		2501.2	506.1	308.0	1674.6
				0.20	*0.12*	*0.67*
	Semi-arid South		2086.1	470.1	241.2	1358.2
				0.23	*0.12*	*0.65*
Rural regions	Arid North		1857.0	369.0	193.4	1292.6
				0.20	*0.10*	*0.70*
	Arid South		1805.3	356.0	168.8	1280.5
				0.20	*0.09*	*0.71*
	Coast		2278.8	472.4	249.3	1556.2
				0.21	*0.11*	*0.68*
	High Rainfall		2313.7	460.8	270.0	1577.5
				0.20	*0.12*	*0.68*

Semi-arid North	2303.0	426.4	281.2	1588.7
		0.19	*0.12*	*0.69*
Semi-arid South	2377.5	465.6	281.6	1623.6
		0.20	*0.12*	*0.68*

The full macro-micro commodity mapping is presented in Annex 7 of Ramos et al. (2020).

Sensitivity analysis of cash transfer measures

The assessment of government measures in the article is complemented by a sensitivity analysis regarding the scale of the household cash transfers as a means to address income reduction. In the base case a total of KSh 10 billion (USD 92 million) is allocated by the government as cash payments to vulenerable households distributed across household groups as described in Table A7.

We seek to assess the implications of both increasing and decreasing the amount of transfers. Considering the large impacts of the base government measures on government deficit (an 89% increase in public deficit in All Measures V-V scenario and 110% in All Measures W-W), we appraise as reasonable a doubling of cash payments (a KSh 10 billion in cash transfers, representing 0.1% of the GDP) which would not put too much additional presure on the public deficit. This step up in transfers is applied as an addition to the All Measures V-V scenario (labeled as All Measures V-V Plus senstivity scenario below). We also assess the implications of a scalling down of the cash payments by reducing these by 50% from the base value (KSh 5 billion on total transfers, labeled as All Measures V-V Minus). It is assumed that in both these new sensitivity analysis scenarios no additional funds from abroad are made available, hence the government covers the corresponding increase in spending through internal borrowing.

The CGE modelling results show that, at a macro-economic level, there is a trade-off between aggregate economic output (measured in GDP, employment and supply) on one hand, and consumer demand and food-related imports on the other (Table A13). An increase in cash transfers (All Measures V-V Plus) leads to a decrease in output but also determines an improvement in consumer demand. Converesely, a decrease in cash transfers (All Measures V-V Minus), improves GDP outcomes but determines a reduction in consumer demand. Neverthless, the differences between the sensitivity analysis scenarios are small since the changes in cash transfers imply a redistribution of internal resources from investment (though government borrowing) to consumption. They represent a small share in the overall GDP, although have a visible impact on government deficit, and are not accompanied by an increase in inflows from abroad in the form of loans or grants. In welfare terms, the scaling up to household transfers benefits mostly the rural households, while the aggregate welfare of peri-urban and metropolitan households marginally decreases due to income effects of lower employment and investment.

For food security, the scaling up of cash transfers determines an increase in aggregate food demand which is translated into higher DEC/capita levels for the bottom and the top quintiles (Figure A5), mainly driven by the increase in DEC/capita in rural areas (Table A14). The lower quintiles that benefit are those households with a calorie intake of below the 2250 calories/capita reference value (see Table A9). For the top income quintiles, the higher effective income in rural areas determines an increase in rural economic activity and consequently an improvement in food consumption of the richer rural households as well. The same trends can be observed in terms of macronutrient intake changes (Figure A6) – the increase in cash transfers determines a higher intake for all three macronutrient types at the extremes of the income distribution. At an aggregate level, the 10 billion KSh increase in cash transfers determines an expansion of households with food sufficiency by 0.1 percentage points and 0.15 percentage points in metropolitan and rural areas respectively, but also a slight reduction of 0.03 percentage points in peri-urban areas.

For a decrase in cash transfers (All Measures V-V Minus), the DEC/capita and macronutrient intakes decrease for lower income quintiles households. For the top income household quintiles, food security metrics do not deteriorate as income levels benefits from lower government spending.

The sensitivity analyis thus illustrates that an increase cash payments to support the declining household income is positive for poorer households, also with positive secondary effects for top quintiles. The negative welfare impact on urban households of an increase in government spending suggest that the scaling up of these transfers should be done by seeking external support in the shape of foreign loans or grants from international donors.

Table A13 – Sensitivity Analysis – Macro-economic results of changes to the cash transfer envelope. Values in percentage point deviations from baseline pre-COVID-19 levels for 2020.

	All Impacts (V-V)	**All Measures (V-V)**	**All Measures (V-V) Plus**	**All Measures (V-V) Minus**	All Measures (V-W)	All Measures (W-W)
Aggregate indicators						
GDP	-5.59	**-4.19**	**-4.22**	**-4.16**	-4.97	-7.85
Employment	-11.99	**-8.05**	**-8.14**	**-7.96**	-10.09	-15.63
Gov deficit	49.72	**88.87**	**91.69**	**86.54**	101.16	110.10
Total supply	-7.01	**-4.12**	**-4.15**	**-4.10**	-6.17	-8.01
Consumer demand	-7.26	**-3.96**	**-3.89**	**-4.01**	-5.80	-8.36
Exports	-8.32	**-8.32**	**-8.32**	**-8.32**	-12.22	-12.22
Imports	-11.62	**-7.01**	**-6.99**	**-7.03**	-12.51	-11.96
Food imports	-14.41	**-9.09**	**-8.89**	**-9.26**	-15.86	-15.32
Welfare indicators (Equivalent Variation)						
Metropolis	-6.96	**-4.20**	**-4.14**	**-4.19**	-6.29	-7.80
Peri-urban	-6.70	**-4.40**	**-4.38**	**-4.43**	-5.86	-8.04
Rural	-8.01	**-5.03**	**-5.19**	**-4.88**	-7.30	-9.07
National	-7.45	**-4.67**	**-4.73**	**-4.60**	-6.72	-8.51

Table A14 – Sensitivity Analysis - Prevalence of food sufficiency and of unbalanced diets across households (% of households). The threshold for food sufficiency is a daily calorific requirement of 2,250 kilocalories. An unbalanced diet implies not meeting at least one of the WHO macronutrient intake proportions.

**HH**	**Baseline**	**All Impacts (V-V)**	**All Measures (V-V)**	**All Measures (V-V) Plus**	**All Measures (V-V) Minus**
**National**	**39.04**	**35.72**	**37.78**	**37.86**	**37.64**
Metropolis	40.08	38.38	40.48	40.58	40.48
Peri-urban regions	41.01	37.64	39.64	39.61	39.65
Rural regions	37.81	34.4	36.48	36.63	36.25
Min HAZ <= -2	20.94	18.41	20.13	20.17	19.87
**National**	**2.73**	**4.41**	**4.40**	**4.40**	**4.39**
Metropolis	6.48	6.38	6.38	6.38	6.38
Peri-urban regions	3.6	5.1	5.08	5.08	5.08
Rural regions	1.93	3.86	3.86	3.86	3.84
Min HAZ <= -2	1.07	3.09	3.05	3.05	3.05

Figure A5 - Change in daily DEC per capita at national level under alternative cash transfer levels (Plus, Minus)

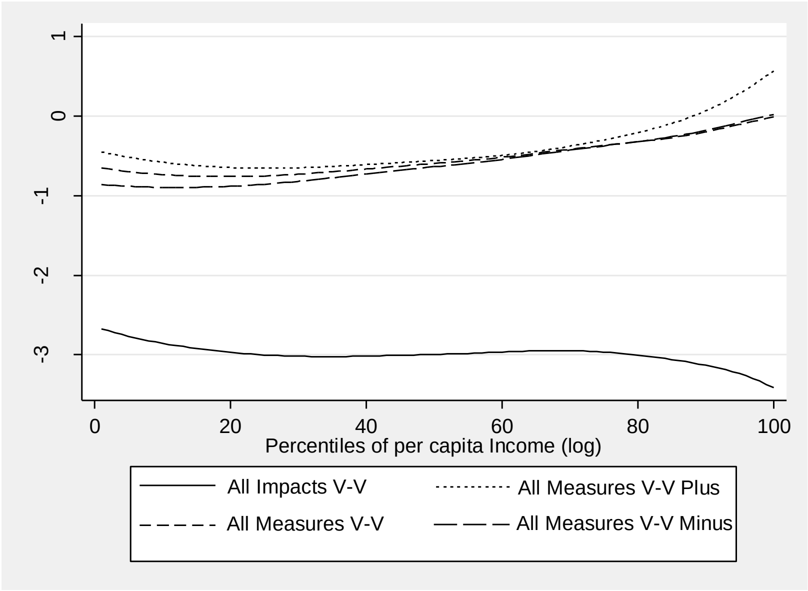

Figure A6 – Sensitivity analysis: Macronutrient intake changes across percentiles of per capita Income under Plus and Minus alternative policy scenarios.

(a) Fat (b) Protein (c) Carbohydrate

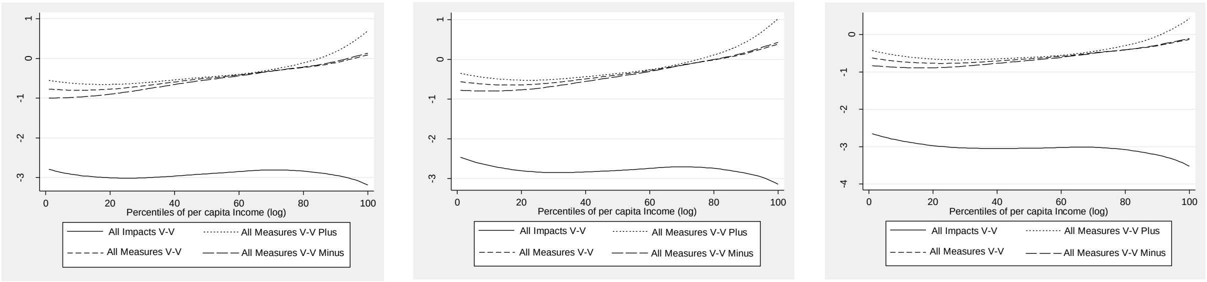

Additional references

KEPSA, 2020a. Accelerating the Pace of Interventions to Address the Impact of Covid-19 on Businesses and the Broader Economy. Kenya Private Sector Alliance, Nairobi.

KEPSA, 2020b. Coordinating private sector efforts and complementing GoK Efforts on the COVID-19 response. Kenya Private Sector Alliance, Nairobi.

KEPSA, 2020c. Flowers of Hope to Save Thousands of Farm Jobs. Kenya Private Sector Alliance, Nairobi.

KNBS, 2020. KNBS Survey on Socio Economic Impact of COVID-19 on Households Report, Wave One. Kenya National Bureau of Statistics, Nairobi.

MoALFC, 2020. Key staples food balance sheet as at end of June 2020.

World Bank, 2020. COVID-19 Crisis Through a Migration Lens. World Bank, Washington, D.C.

WTO, 2020. Methodology for the WTO Trade Forecast of April 8 2020. World Trade Organization, Economic Research and Statistics Division.
